# Polymyxins: recent advances and challenges

**DOI:** 10.3389/fphar.2024.1424765

**Published:** 2024-06-21

**Authors:** Shan Yang, Hairui Wang, Dan Zhao, Shurong Zhang, Chenggong Hu

**Affiliations:** ^1^ Department of Critical Care Medicine, West China Hospital of Sichuan University, Chengdu, Sichuan, China; ^2^ Institute of Respiratory Health, West China Hospital of Sichuan University, Chengdu, Sichuan, China

**Keywords:** antibacterial mechanism, drug resistance mechanisms, nanotechnology, polymyxins, pharmacokinetics

## Abstract

Antibiotic resistance is a pressing global health challenge, and polymyxins have emerged as the last line of defense against multidrug-resistant Gram-negative (MDR-GRN) bacterial infections. Despite the longstanding utility of colistin, the complexities surrounding polymyxins in terms of resistance mechanisms and pharmacological properties warrant critical attention. This review consolidates current literature, focusing on polymyxins antibacterial mechanisms, resistance pathways, and innovative strategies to mitigate resistance. We are also investigating the pharmacokinetics of polymyxins to elucidate factors that influence their *in vivo* behavior. A comprehensive understanding of these aspects is pivotal for developing next-generation antimicrobials and optimizing therapeutic regimens. We underscore the urgent need for advancing research on polymyxins to ensure their continued efficacy against formidable bacterial challenges.

## 1 Introduction

**FIGURE 1:**
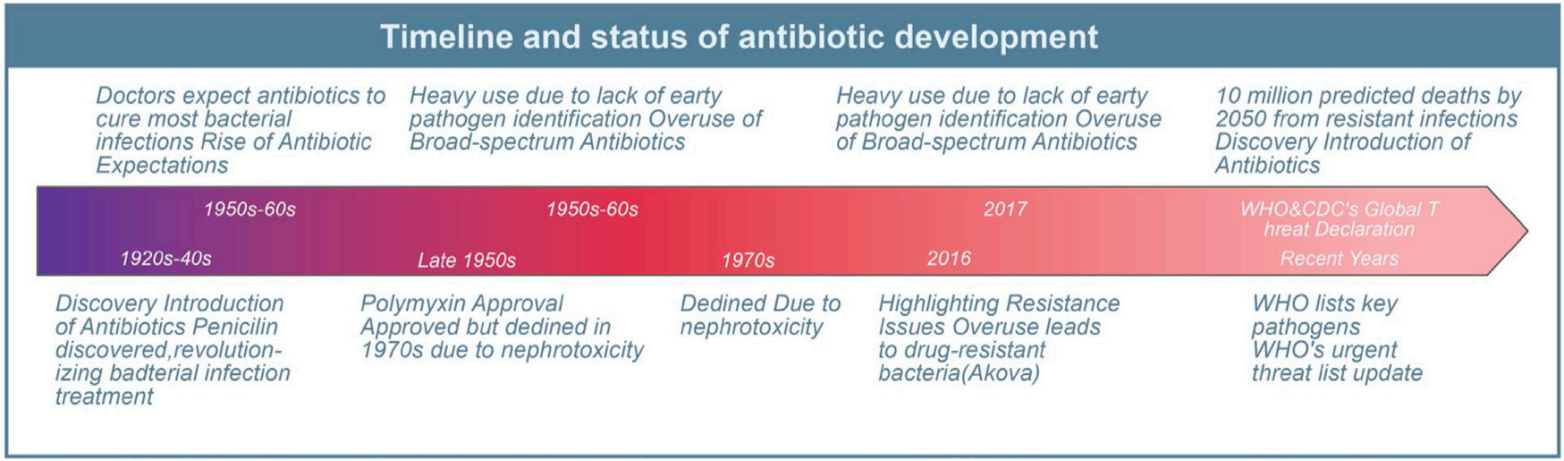
Antibiotic classification and current status of clinical resistance. This diagram illustrates the evolution and challenges of antibiotic use since its inception.

Since the introduction of antibiotics in the last century, they have saved countless lives of patients with serious bacterial infections. In the past 50–60 years, doctors have come to expect that antibiotics would cure almost all patients with bacterial infections. However, due to the lack of early identification of the causative organisms and their antimicrobial susceptibility patterns in patients with bacteremia and severe infections in many healthcare facilities, broad-spectrum antibiotics have been heavily and mostly unnecessarily used since the 1990s (Akova, 2016). Consequently, this has led to the emergence of numerous drug-resistant bacteria and unregulated management of nosocomial infections, resulting in increased chances of transmission of drug-resistant bacteria, longer hospital stays, and higher mortality rates for patients (Akova, 2016). In 2017, the World Health Organization (WHO) added *Acinetobacter* baumannii, *Pseudomonas aeruginosa*, and *Enterobacter* species to the list of key pathogens in urgent need of new antibiotics (Mancuso et al., 2021). While medical institutions have conducted certain control and preliminary systematic evaluations of these pathogens (Tomczyk et al., 2019), the overuse and misuse of antibiotics in healthcare, agriculture, and livestock have contributed to a significant increase in antimicrobial resistance (Nadimpalli et al., 2020; Schrader et al., 2020). WHO and the U.S. Centers for Disease Control and Prevention (CDC) have recognized antimicrobial resistance as a worldwide threat. Without effective management and scaling up the supply of antibiotics, nearly 10 million people worldwide are expected to die from drug-resistant infections by 2050 (Pulingam et al., 2022). Although the U.S. Food and Drug Administration (FDA) has approved several new antibiotics in recent years, the emergence of resistance has been reported (Abdallah et al., 2015; Giddins et al., 2018; Morrissey et al., 2020) (Figure 1). Therefore, there is an urgent global need for antimicrobials with innovative pharmacological activities and modes of action to combat the public health threat of antimicrobial resistance (Miethke et al., 2021).

Polymyxins have received increasing attention in recent years, as they are considered a potential weapon against Gram-negative drug-resistant bacteria. Polymyxin was approved in the late 1950s (Li et al., 2006). However, its usage rapidly declined in the 1970s due to its nephrotoxicity. Nevertheless, with carbapenem-resistant *Acinetobacter* baumannii (CRAB), *Pseudomonas aeruginosa*, and *Klebsiella pneumoniae* resistance rates increasing each year (Nang et al., 2021), polymyxins have been reintroduced into the clinic as a last-resort salvage treatment option for these resistant organisms (Cai et al., 2012; Doi et al., 2015). Over the past two decades, significant progress has been made in the study of polymyxins, including their chemical structure, activity/toxicity relationship, antimicrobial activity, and polymyxin resistance mechanisms. This review summarizes the history of polymyxin development and provides an overview of the mechanisms of drug resistance. Additionally, it focuses on the research conducted to overcome colistin resistance and highlights the development of new antimicrobials that have entered clinical trials. Furthermore, the review presents the latest research progress in overcoming polymyxin resistance and sheds light on the pharmacokinetic behavior of polymyxins to improve the standardization and safety of their global clinical application.

## 2 Polymyxins: from discovery to re-emergence in the era of superbugs

**FIGURE 2:**
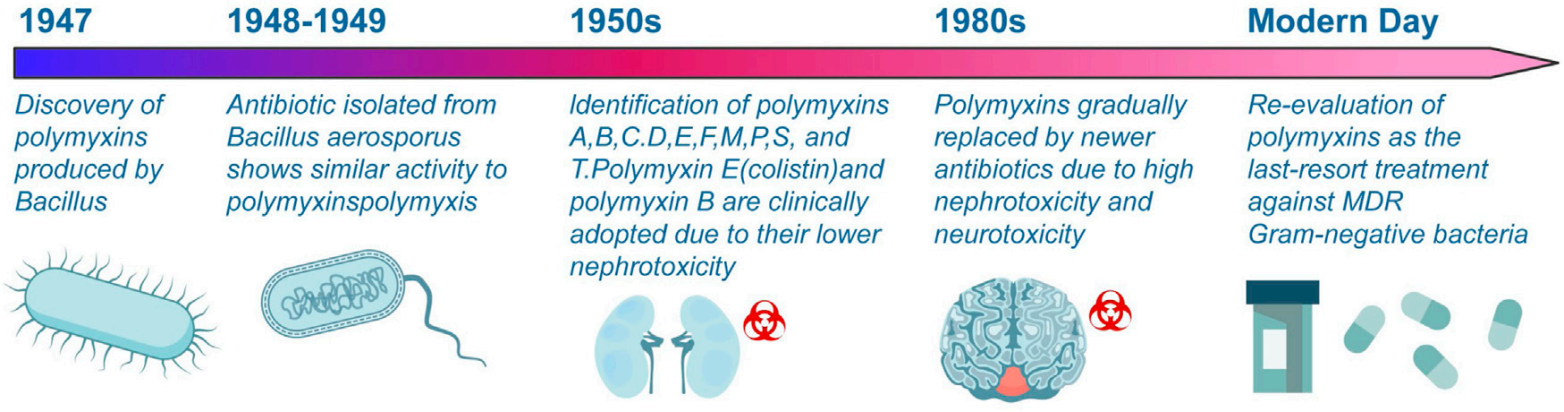
Evolution of polymyxin antibiotics. This illustration delineates the significant milestones in developing polymyxin antibiotics, from their discovery in 1947 to their contemporary relevance.

Polymyxins, discovered in 1947, are antimicrobial cationic polypeptides produced by *Bacillus* polymyxins (Benedict and Langlykke, 1947). Figure 2 provides a timeline highlighting the major milestones in the discovery, use, and resurgence of polymyxins. Brownlee and Bushby (1948) isolated an antibiotic from *Bacillus aerospore* that exhibited antibacterial activity against Gram-negative bacteria. In 1949, White et al. conducted a comparative study on the antibacterial activities of polymyxins and “Neosporin” and found no significant difference between them, suggesting that both substances belong to the polymyxins class of antibiotics. Consequently, a nomenclature system was established for the polymyxins family (Stansly and Brownlee, 1949). To date, polymyxin A (also known as Neosporin), B, C, D (polymyxin), E (also known as colistin), F, M, P, S, and T have been identified from *P. Polymysa* strains (Shoji et al., 1977; Niu et al., 2013). After their discovery, many polymyxins were found to have reversible nephrotoxicity, leading to the clinical use of colistin and polymyxin B due to their relatively low nephrotoxicity. The main market products are polymyxin B and polymyxin E, which exhibit similar antimicrobial activity (Storm et al., 1977). There are currently three polymyxin analogues for injection that have been marketed both domestically and internationally: colistin methanesulfonate (CMS) for injection, colistin sulfate and polymyxin B sulfate for injection. The blood-brain barrier passage rate of polymyxin is low, and it is difficult to achieve effective drug concentration by intravenous administration. Local applications such as intracerebroventricular or intrathecal administration have been increasingly adopted by the clinic in recent years, but there is a lack of relevant standardized operational guidelines (Yang et al., 2023). In addition, the nebulized inhalation method can significantly increase the lung tissue concentration of polymyxin while decreasing the systemic exposure level of the drug, thus achieving the goal of improving the efficacy and reducing systemic adverse effects (Tang et al., 2023). Currently, several domestic and international guidelines and consensus recommend nebulized inhalation of polymyxin as one of the important therapeutic methods for multidrug-resistant gram-negative (MDR-GRN) bacterial induced pneumonia (Lin et al., 2022).

**FIGURE 3:**
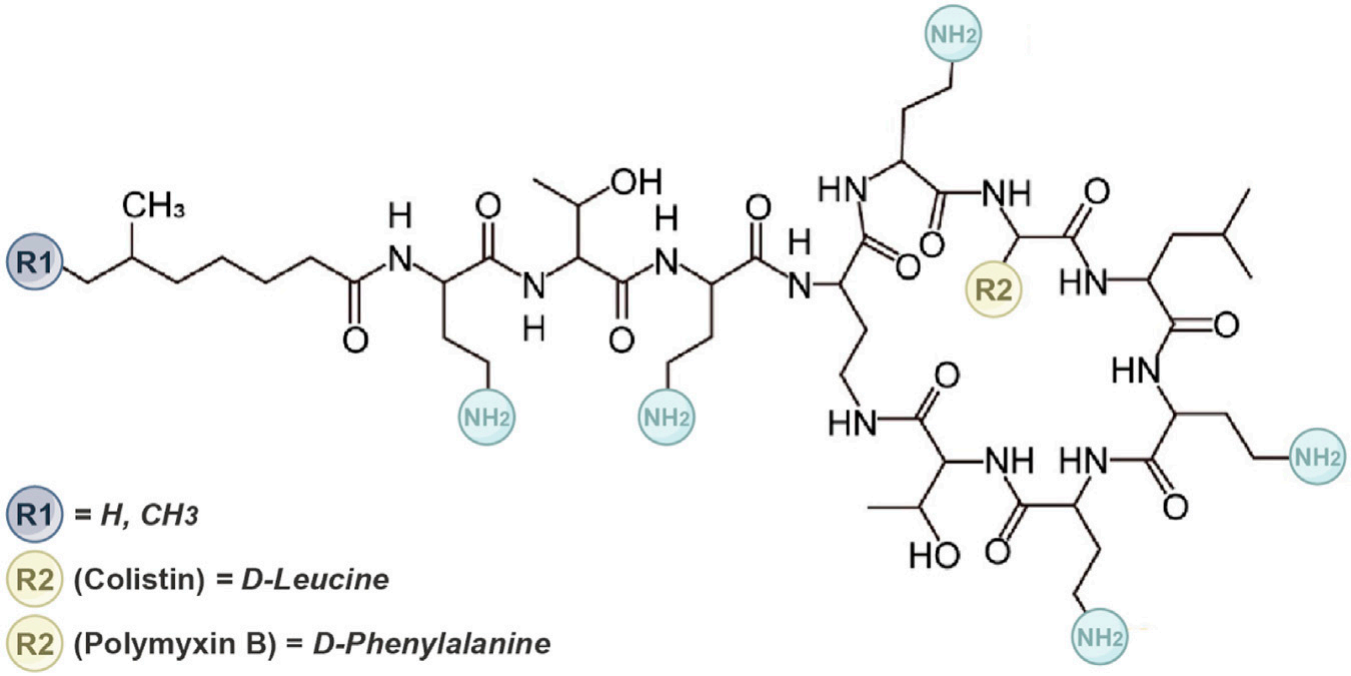
The molecular structure of the polymyxin. It outlines the structural distinctions between polymyxin B and colistin. Polymyxin B and colistin comprise five conserved L-α-γ-diaminobutyric acid (Dab) residues. These residues confer a net positive charge to polymyxin compounds at physiological pH. The cationic hydrophilicity of this macrocycle is pivotal for their antibacterial properties.

Polymyxin is a cyclic lipopeptide compound composed of 10 amino acids, characterized by a cationic polypeptide consisting of a cyclic heptapeptide and a tripeptide side chain (Rutten et al., 1990; Falagas et al., 2010). The primary difference between PMB and colistin lies in the amino acid variation at position 6 (R2), where PMB contains phenylalanine and colistin contains leucine (Figure 3) (Velkov et al., 2010; Nation et al., 2014). They have similar antimicrobial effects. Compared to the parent antibiotics, sulfomethyl derivatives of polymyxins exhibit lower toxicity and similar *in vivo* antibacterial activity. Consequently, sulfonated derivative polymyxin E has been commercially available for clinical use in Japan, Europe, and the United States since the 1950s. Conversely, colistin methanesulfonate sodium, an antibacterial active component covered by sulfate, lacks inherent antibacterial activity and serves as a precursor drug that exhibits bactericidal effects. However, CMS was gradually replaced by newer antibacterial drugs in the 1980s due to its high nephrotoxicity and neurotoxicity. Importantly, commercial polymyxin B, CMS, and colistin products are mixtures (Govaerts et al., 2002), leading to batch-to-batch differences in the abundance of individual ingredients.

With limited antibiotic options available, the increasing bacterial resistance in clinical settings has necessitated the re-evaluation of “old” antibiotics, particularly polymyxin, which has shown effectiveness against many MDR Gram-negative bacteria. Polymyxins have been used in clinical practice for approximately 60 years, with polymyxin B and colistin now considered last-resort treatment options for infections caused by “superbugs."

## 3 Deciphering the multimodal antibacterial strategies of polymyxins

Understanding the mode of action of polymyxins is crucial for optimizing their use and developing new antibiotics. Polymyxin B and colistin, having similar chemical structures, exhibit comparable antibacterial mechanisms primarily against common Gram-negative bacteria (Kwa et al., 2007). Colistin demonstrates a narrow spectrum of antibiotics but shows activity against several clinically significant MDR Gram-negative bacteria, such as *Pseudomonas aeruginosa*, *Acinetobacter baumannii*, *Escherichia coli*, *Klebsiella pneumoniae*, *Enterobacter*, and other enterobacteriaceae (Bialvaei and Samadi Kafil, 2015; Poirel et al., 2017; Doymaz and Karaaslan, 2019).

**FIGURE 4:**
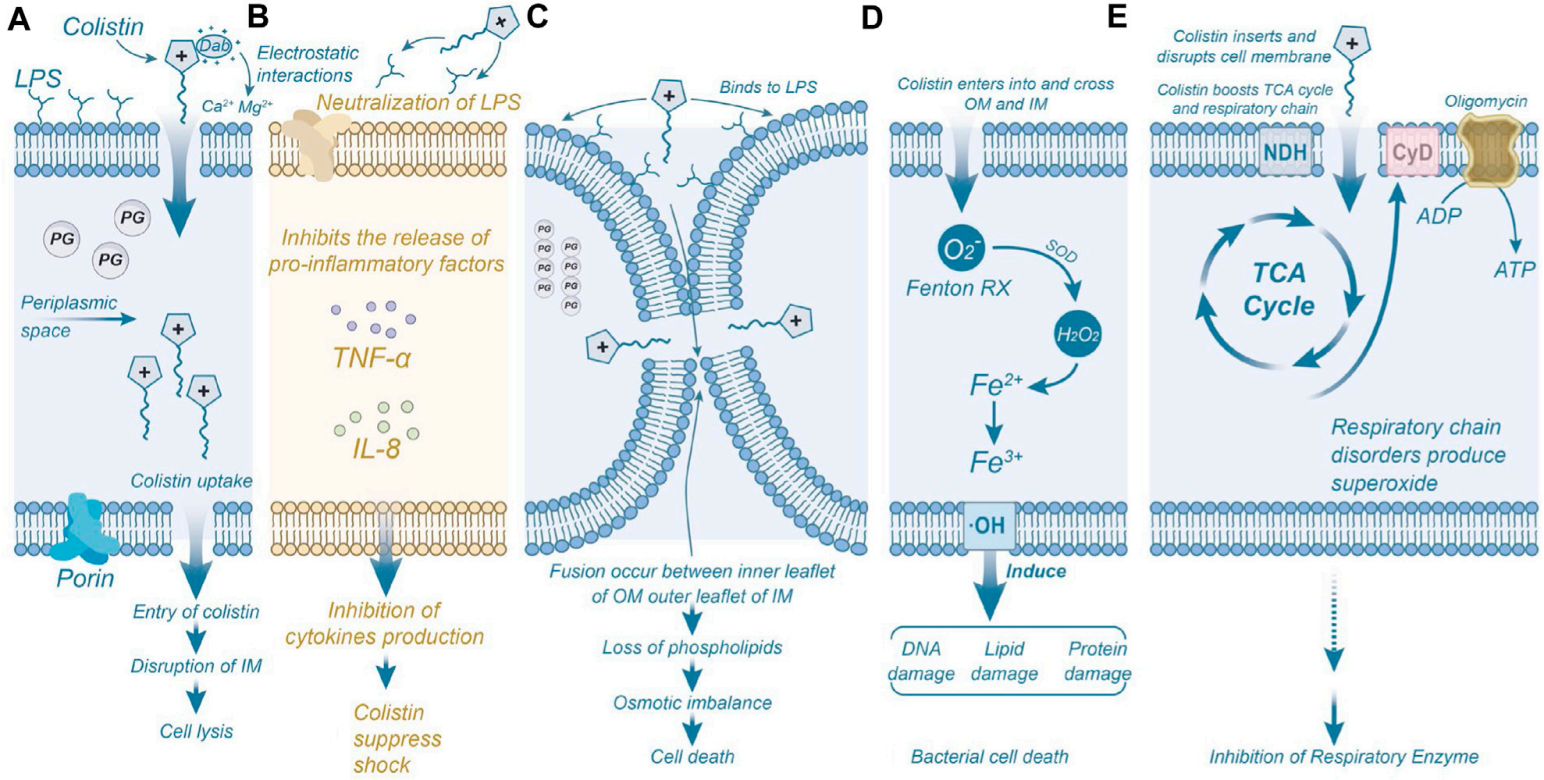
Mechanisms of Polymyxins-Induced Bacterial Inhibition and Death. **(A)** The Dab residue of colistin interacts electrostatically with the anionic phosphate group in the outer membrane of bacteria, resulting in structural disruption of the bacterial membrane. **(B)** Colistin neutralizes the activity of LPS molecules, inhibiting the induction of shock and consequent release of cytokines by immune cells, such as tumor necrosis factor-α and interleukin 8. **(C)** Colistin binds to phospholipids on the outer membrane of bacteria, causing depletion of phospholipids and leading to bacterial death. **(D)** Colistin triggers the production of reactive oxygen species (ROS) that damage DNA, lipids, and proteins, ultimately resulting in bacterial death. **(E)** Colistin inhibits the activity of respiratory enzymes. Permission to reproduce adapted from reference (El-Sayed Ahmed et al., 2020) has been obtained.

Both colistin and polymyxin B exert their effects on the bacterial membrane, causing damage. The outer membrane of Gram-negative bacteria serves as protection against various harmful substances, including antimicrobials. Extensive studies have demonstrated that polymyxins exerts its antibacterial effect by directly interacting with the lipid components of lipopolysaccharide (LPS), disrupting bacterial membranes (Nikaido, 2003). Electrostatic interactions occur between the positively charged α, γ-aminobutyric acid (Dab) residues of polymyxin and the phosphoric acid groups on the bacterial membrane, competitively displacing divalent cations (Ca^2+^ and Mg^2+^) through the negatively charged phosphoric acid groups in the lipid membrane (Dixon et al., 1986). In addition, polymyxins can neutralize endotoxins and inhibit the expression of cytokines such as TNF-α and IL-8, preventing tissue damage caused by excessive activation of inflammation (Schromm et al., 2021). Polymyxins binds to LPS through electrostatic and hydrophobic interactions (Velkov et al., 2010). Consequently, LPS becomes unstable, increasing bacterial membrane permeability, leading to the leakage of cytoplasmic contents and, ultimately, bacterial death (Schindler and Osborn, 1979; Falagas and Kasiakou, 2005). While the primary model for polymyxin’s antimicrobial activity involves the destruction of the bacterial adventitia and intima, additional mechanisms have also been proposed (Figure 4).

Another mechanism of polymyxin action is the inhibition of type II NADH-quinone oxidoreductase (NDH-2) activity, a significant respiratory enzyme in the bacterial inner membrane (Deris et al., 2014). After entering the bacteria, polymyxin inhibits the respiratory enzymes of the tricarboxylic acid (TCA) cycle and consumes ATP, leading to bacterial death. Studies have demonstrated that polymyxins inhibits NDH-2 activity in a concentration-dependent manner in various Gram-negative bacteria, including *Escherichia coli*, *Klebsiella pneumoniae*, and *Acinetobacter baumannii* (Mogi et al., 2009). These events are accompanied by activation of repair pathways and adventitial remodeling (Moffatt et al., 2019). Additionally, some researchers propose that most antibiotics induce bacterial death by perturbing bacterial metabolism, leading to increased production of reactive oxygen species (ROS), including superoxide (O_2_^−^), hydrogen peroxide (H_2_O_2_), and hydroxyl radicals (-OH), ultimately causing bacterial demise (Kohanski et al., 2007). Leveraging this property, many researchers have designed antimicrobial agents that promote bacterial death by stimulating the production of hydroxyl radicals through the Fenton reaction (Yeom et al., 2010). Elevated levels of hydroxyl radicals within bacteria can damage bacterial DNA, lipids, and protein synthesis, resulting in bacterial death (Dwyer et al., 2014). These findings indicate the importance of hydrophobic interaction in the antibacterial mechanism of polymyxin (Hancock, 1997).

## 4 Unraveling the complex web of polymyxin resistance: mechanisms, challenges, and countermeasures

The escalating issue of antibiotic resistance poses a significant threat to human health, particularly with the emergence of resistance in *Enterobacteriaceae*, *Acinetobacter baumannii*, and *Pseudomonas aeruginosa* against almost all available antibiotics. Such MDR bacteria are commonly found in intensive care units and among long-term hospitalized patients. Despite their potent bactericidal activity against many Gram-negative bacteria, the extensive use of polymyxins has led to the emergence of polymyxin-resistant strains, including *Neisseria meningitidis*, *Proteus mirabilis*, and *Burkholderia s*pp. (Tzeng et al., 2019). Notably, nosocomial infections caused by MDR *Pseudomonas aeruginosa*, *Acinetobacter baumannii,* and *Klebsiella pneumoniae* are closely associated with increased morbidity and mortality, and their resistance to polymyxins has attracted significant attention (Barbier et al., 2013; Bassetti et al., 2018).

**FIGURE 5:**
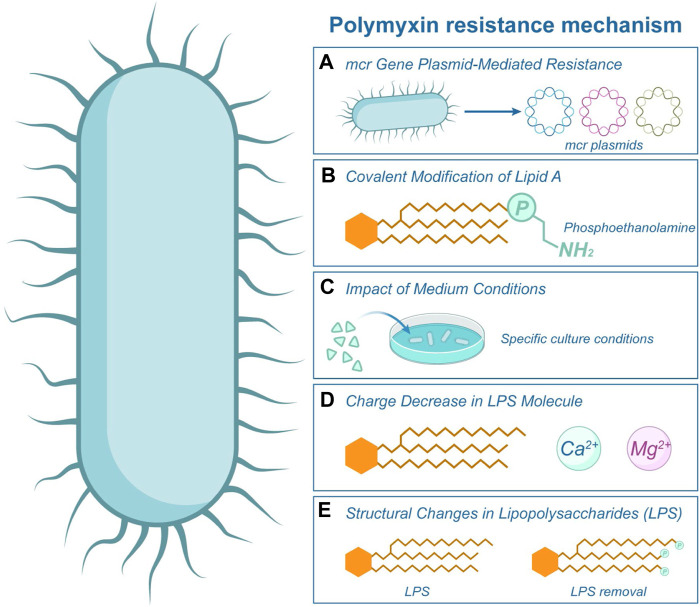
Polymyxin Resistance Mechanism. This illustration showcases the various mechanisms by which bacteria develop resistance to polymyxin. Central to this resistance is the structure of Lipopolysaccharide (LPS) present in the bacterial cell, as highlighted in figure. **(A)** The *mcr* gene on bacterial plasmids facilitates the primary resistance mechanism, as depicted by the label *mcr* in the image. Furthermore, resistance can arise from covalent modifications to Lipid A **(B)** or structural alterations in the LPS **(E)**. The efficacy of polymyxin is further diminished by the reduced charge on LPS **(D)**, especially when it interacts with cations such as Mg^2+^. **(C)** The growth medium conditions in which bacteria reside can also influence their susceptibility to polymyxin.

In this section, we present a systematic review of recent discoveries on the mechanisms of polymyxin resistance. In recent years, antibiotic resistance genes have become one of the greatest threats to human health in the 21st century. *mcr*-1, the first plasmid-mediated polymyxin resistance gene, was discovered in China in 2015, indicating that the last line of defense has also been breached, further exacerbating the threat of bacterial resistance to public health. Table 1 summarized the resistant bacteria and resistance genes to polymyxins in recent years. Some of the more attention-grabbing, classical mechanisms of polymyxin resistance are summarized in Figure 5. The phosphoethanolamine (pEtN) transferase encoded by *mcr*-1 delivers pEtN from the cell membrane through modification of lipid A, leading to colistin resistance (Figure 5A). This modification reduces the affinity of polymyxin for lipopolysaccharides, as depicted in Figure 5B (Needham and Trent, 2013; Liu et al., 2016). In addition to colistin resistance, *mcr* mediates bacterial resistance to antimicrobial peptides (AMPs). The amphiphilic structure of AMPs enables them to penetrate bacterial cell membranes, forming pores and disrupting the integrity of the cell membrane, leading to lysis and death of the bacteria (Nang et al., 2021; Rodriguez-Santiago et al., 2021). The emergence of the plasmid-mediated colistin resistance gene *mcr*-1 has attracted global attention and prompted several countries to adjust their policies on the use of colistin in food and animals. Currently, researchers have conducted extensive studies on the distribution, function, mechanism of action, transmission vectors, and origin of *mcr*, as well as prevention and control strategies for *mcr*-positive bacteria (Liu et al., 2024). Some researchers have recently identified the problem of polymyxin resistance mediated by multiple *mcr* gene plasmids (Liu et al., 2016; Schwarz and Johnson, 2016). Certain environments, such as hospitals with a high potential for transmission of resistant bacteria through food or surfaces, as well as heavily contaminated surface water, can serve as reservoirs for various infections.

**TABLE 1:** Bacteria resistant to polymyxins and their resistance gene.

Bacteria	Resistance mechanisms	Gene	References
*A. baumannii*	Modifications of the LPS moiety	*pmr*A, *pmr*B, *pmrC*, *mcr*	Snitkin et al. (2013), Cheah et al. (2016), Lima et al. (2018)
*A. baumannii*	lipid A deficiency	*lpx*A, *lpx*C, and *lpx*D	Bojkovic et al. (2015), Jeannot et al. (2017), Sherry and Howden (2018)
A. baumannii	Changes in membrane permeability and Efflux pump systems	*lps*B, *lpt*D, and *vac*J	Hood et al. (2013), Whitfield and Trent (2014), Thi Khanh Nhu et al. (2016)
*Helicobacter pylori*	Modifications of the LPS moiety	*Cgt, ompD*	Sato et al. (2018)
*Neisseria meningitidis*	Efflux pumps	*por*B	Tzeng et al. (2005)
*Yersinia enterocolitica*	RosA/RosB efflux pump/potassium antiporter system	*RosA* and *RosB*	Bengoechea and Skurnik (2000)
*Pa. polymyxa*	Inactivates colistin	Unknown	Sampson et al. (2012), Deris et al. (2014)
*P. mirabilis*	LPS modification	*Proteus* *pmrI* gene	Jiang et al. (2010)
*S. marcescens*	LPS modification	*arn*B and *arn*C	Lin et al. (2014)
*K. pneumoniae*	Overexpression of PhoP/PhoQ, Inactivation of the *mgrB* gene	ColR/ColS, *blaCTX-M-15 ESBL* gene	Gutu et al. (2013), Cannatelli et al. (2014), Jayol et al. (2015a), Jayol et al. (2016), Nordmann et al. (2016)
*Klebsiella pneumoniae*	Reduces the affinity of antibiotics for cells	*mgrB* gene	Jayol et al. (2015b), Yap et al. (2022)
*Aeromonas spp* and *Escherichia coli*	*mcr*-variant of the plasmid-mediated	*mcr*-variant	Belaynehe et al. (2018), Gonzalez-Avila et al. (2021)
*P. aeruginosa*	LPS modification operon	*ParR/ParS*	Fernandez et al. (2010)
*V. cholera*	LPS biosynthesis and modification	*gspIEF, lpxN, vc0224/0239/1981*	Mlynarcik and Kolar (2019)
*Haemophilus influenza*	Lipooligosaccharide modifications	*lic1/2A, lpsA, lgtF, opsX*	Morey et al. (2013)

It has also been observed that the conditions of the medium influence the sensitivity of some bacteria to polymyxins. *Salmonella enterica* cells lacking carbon, nitrogen or phosphate ions, and serum and quiescence cells exhibit reduced sensitivity to polymyxin (Figure 5C) (McLeod and Spector, 1996). An alternative mechanism involves the activation of the *pmrA* genes in *Salmonella typhimurium*, triggered by low levels of Mg^2+^ ions. It decreases the lipopolysaccharide molecule’s negative charge and subsequently reduces polymyxin’s binding affinity (Groisman et al., 1997), as depicted in Figure 5D.

Previous studies have demonstrated that the resistance mechanism in most Gram-negative bacteria is associated with structural changes in lipopolysaccharides (Moore et al., 1984; Pelletier et al., 2013). Bacteria can develop resistance to polymyxin either by modifying the phosphate group of lipid A or by directly removing LPS (Moffatt et al., 2010; Olaitan et al., 2014), as depicted in Figure 5E. For instance, in the presence of high concentrations of polymyxin in *Pseudomonas aeruginosa*, the bacterial acidic phospholipids undergo conversion to neutral lipids, along with changes in proteins and carbohydrates, ultimately leading to the development of resistance (Gilleland and Lyle, 1979; Gilleland and Farley, 1982). Zhao et al. found that the increase of polymyxin concentration would affect the dynamics of genetic variants in the flora and lead to different degrees of evolution of resistance, and emphasized that during the use of polymyxin, the evolutionary findings were integrated into pharmacokinetics/pharmacodynamics to improve the antibacterial efficacy of patients (Zhao et al., 2022).

Antibiotic resistance has significantly compromised the effectiveness of antibiotics, leading to a substantial burden on medical care improvement and cost control. To address this issue, healthcare professionals must use antibiotics judiciously, preventing misuse and overuse, which can delay the emergence of resistance and reduce healthcare expenses for patients. Governments and medical institutions should also manage and optimize antibiotic use patterns, selecting the most suitable treatment plan based on recommended dosages and durations to achieve optimal clinical outcomes while minimizing toxicity and the risk of subsequent resistance. Furthermore, establishing an integrated and specialized antibiotic use monitoring system can help detect and prevent the emergence of antibiotic resistance in advance, addressing the problem at its source. Pharmaceutical researchers, in particular, should intensify their efforts in developing polymyxin antibiotics to counter emerging or potentially resistant bacteria in the future.

## 5 Innovative strategies in combating antibiotic resistance

Antibiotic resistance poses a significant challenge to global health, necessitating the exploration of diverse strategies to counteract its proliferation. Yan Zhu et al. proposed the combination of genome-scale metabolic modeling with multi-omics data elucidated the mechanisms by which A. baumannii cells respond to colistin treatment, including (i) upregulation of gluconeogenesis, pentose phosphate pathway, amino acid, and nucleotide biosynthesis fluxes; (ii) downregulation of TCA cycling, peptidoglycan, and lipopolysaccharide biogenesis; and (iii) alterations in respiratory chain fluxes. The findings elucidate the interaction of multiple metabolic pathways in A. baumannii when treated with colistin and provide key mechanistic insights for optimizing polymyxin combination therapy (Zhu et al., 2019). Li J et al. revealed the key pathways associated with the synergistic activity of polymyxin B and rifampicin in combination against multidrug-resistant *Acinetobacter baumannii* by comparative metabolomics. They found that polymyxin B monotherapy significantly disrupted glycerophospholipid and fatty acid metabolism within 1 h, reflecting its activity against the bacterial outer membrane. Rifampicin monotherapy significantly disrupted glycerophospholipid, nucleotide, and amino acid metabolism, which was associated with inhibition of RNA synthesis, and with the combination, polymyxin B initially affected pathways associated with outer membrane biogenesis, whereas rifampicin affected them through inhibition of RNA synthesis, and the findings provide new mechanistic insights into optimizing this synergistic combination in patients (Zhao et al., 2021). As bacteria continue to evolve and resist conventional treatments, the scientific community has responded with vigor, delving deep into combination therapies, chemobiological innovations, and the potential of nanotechnology.

### 5.1 Polymyxin combination therapies: overcoming bacterial resistance

Combining polymyxin with sensitizing drugs represents a promising strategy to overcome polymyxin resistance and restore its sensitivity. Srisakul et al. proposed the combination of polymyxin and sulbactam as a means to overcome lipid A-mediated colistin resistance (Srisakul et al., 2022). Moreover, Chen et al. reported that Anthranilyl-CoA Synthetase PqsA effectively enhanced the activity of polymyxin B against MDR *Pseudomonas aeruginosa*-associated infections (Chen J. et al., 2022). In a study by Li et al., it was demonstrated that the combined use of the guanidine derivative isopropoxy benzene guanidine with low-level colistin enhanced the permeability of the bacterial outer membrane and increased the accumulation of reactive oxygen species, thereby combating MDR *Escherichia coli* (Li et al., 2023). Shein et al. suggested combining colistin and EDTA could overcome *mgrB*-mediated colistin resistance in carbapenem-resistant *Klebsiella pneumoniae* (Shein et al., 2022). Wang et al. showed that combination therapy using colistin and resveratrol improved the membrane permeability of bacteria and increased the sensitivity of *Pseudomonas aeruginosa* to colistin (Wang et al., 2023). The plasmid-mediated resistance gene *mcr*-1, a homolog of eptA, confers resistance by modifying lipid A through cationic phosphoethanolamine (Liu et al., 2016). MacNair et al. (2018) demonstrated that combining polymyxin with antibiotics targeting Gram-positive bacteria effectively treated infections caused by drug-resistant Gram-negative bacteria expressing *mcr*-1 (MacNair et al., 2018).

Additionally, combination therapy using melatonin and colistin has shown efficacy in eradicating *mcr*-positive pathogens and exhibits a favorable biosafety profile. The combined antibacterial mechanism of polymyxin and melatonin involves enhancing bacterial outer membrane permeability, promoting oxidative damage, and inhibiting the expression of bacterial efflux proteins. Liu et al. demonstrated that the combination of polymyxin and melatonin significantly restored the efficacy of colistin in three animal models of *E. coli* infection carrying *mcr*-1 (Liu et al., 2020). Table 2 presents an overview of clinical trials investigating polymyxin combination therapy to overcome drug resistance. Lindsey A Carfrae et al. proposed that the biotin biosynthesis inhibitor MAC13772 acted synergistically with colchicine, indirectly disrupting fatty acid synthesis (FAS) via MAC13772, leading to changes in phospholipid composition and restoring susceptibility to the antibiotic colchicine. In addition, the investigators propose that combination therapy using colchicine and the clinically relevant FabI inhibitor Debio1452-NH_3_ is efficacious against systemic infections in mice with *mcr*-1-positive *Klebsiella pneumoniae* and colchicine-resistant *Escherichia coli*. We explored the mechanism of this interaction using chemogenomics, lipidomics, and transcriptomics (Carfrae et al., 2023).

**TABLE 2:** Clinical trial of polymyxin involved in drug resistance.

Status	ClinicalTrials.gov identifier	Condition or disease	Drug	Combination of drugs
Phase 3	NCT03159078	Trauma	Polymyxin B	Carbapenem
		Resistant Infection		
		Critical Illness		
Phase 3	NCT02134106	Bacteremia	Polymyxin B	Doripenem
		Healthcare-associated Pneumonia		
		Ventilator-associated Pneumonia		
Completed	NCT00753558	Carriage of Carbapemen-resistant *Klebsiella pneumoniae*	polymyxin E	Oral solution and buccal gel of gentamicin
Recruiting	NCT04839653	Respiratory Tract Infections	polymyxin B	Gentamycin, amphotericine B
		Critical Illness		
Phase 4	NCT04489459	Blood Stream Infections Due to MDR *Klebsiella pneumoniae*	Colistin	Meropenem, Tigecycline
Completed	NCT01266499	*Klebsiella Pneumoniae* Carbapenemase Resistant Associated Bacteremia or Pneumonia	Colistin	Garamycin
Early Phase 1	NCT03950544	Antibiotic-Resistant Infection	Polymyxin B	Fosfomycin, Tigecycline
Recruiting	NCT04202861	Antibiotic Therapy	Polymyxins	Antibiotic combination
Phase 3	NCT05258851	Carbapenem-Resistant Enterobacteriaceae Infection	Colistin	Ceftazidime-avibactam
Phase 2	NCT02472600	Intestinal Colonization With MDR Bacteria	Colistin	Neomycin; Fecal microbiota transplantation; Omeprazole

### 5.2 Chemobiological advancements: enhancing antimicrobial efficacy against drug-resistant pathogens

Jonathan M. Stokes et al. found that pentamidine and its structural analogues sensitize Gram-negative pathogens to antibiotics and overcome acquired resistance to polymyxins (Stokes et al., 2017). Velkov and Li et al. optimized the structure of polymyxin by chemical biology and successfully developed a new lipopeptide, which has significant antibacterial activity against a variety of drug-resistant pathogens. The antimicrobial peptide has entered phase I clinical trials (Roberts et al., 2022). Zsolt Szűcs et al. prepared a vancomycin polycationic glycogen derivative with an n-decane side chain and 5 aminoethyl groups, which has a structure similar to that of polymyxin and can act synergistically against *Escherichia coli*, *Pseudomonas aeruginosa*, and *Acinetobacter baumannii* by combining with teicoplanin (Szucs et al., 2022). Lindsey A. Carfrae et al. have found that the sensitivity of colistin therapy can be restored by inhibiting the synthesis of fatty acids in bacteria and finally changing the lipid composition of bacterial membranes (Carfrae et al., 2023).

### 5.3 Harnessing nanotechnology: a frontier in optimizing antibiotic delivery and performance

**FIGURE 6:**
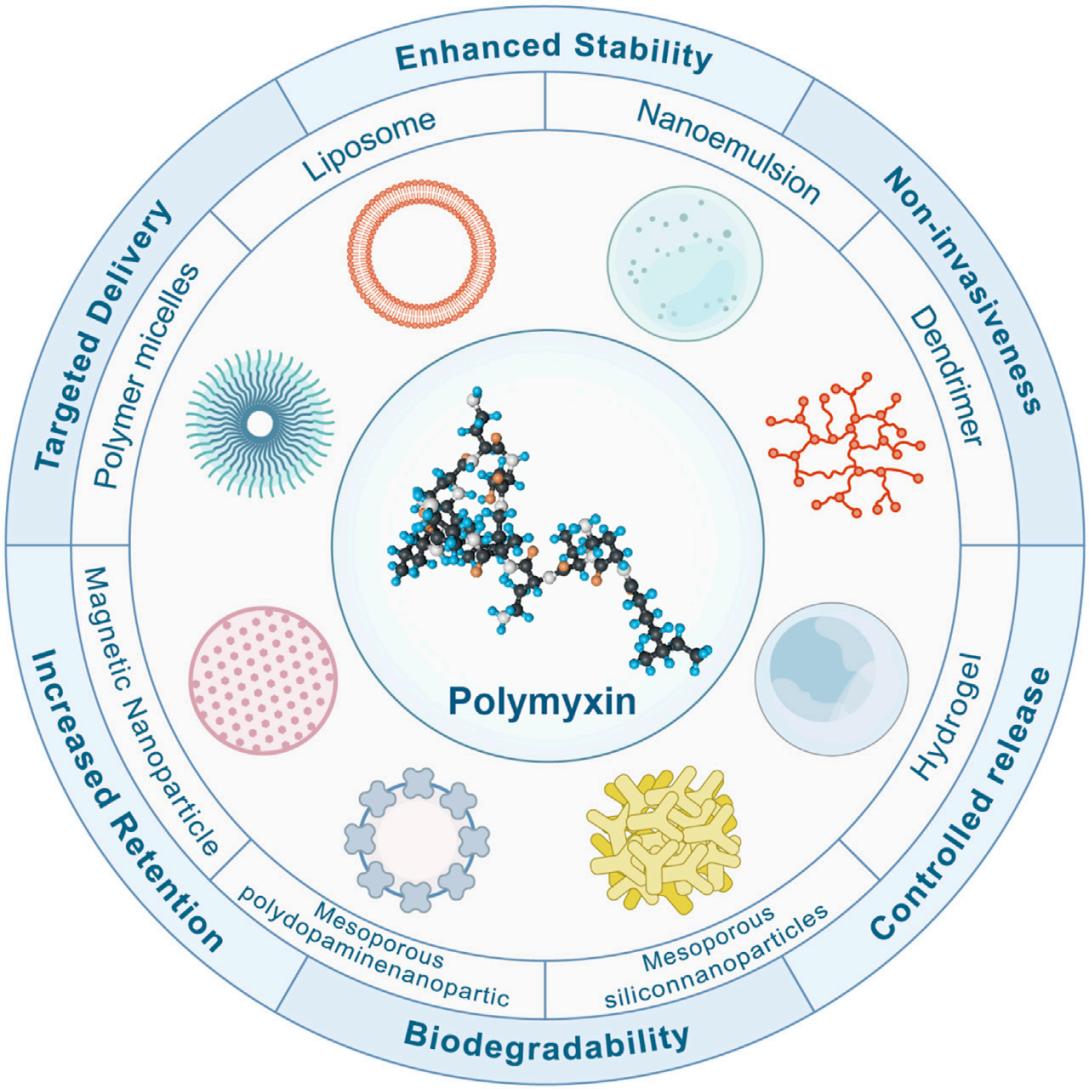
Features of Nanotechnology in Improving Polymyxin Resistance. Nanotechnology has emerged as a cornerstone in the battle against antibiotic resistance. Nano-carriers bolster the resilience of antibiotics amidst the intricacies of physiopathological landscapes. Harnessing the potential of gels and polymer nanofibers, the longevity of antibiotic release is notably augmented. Moreover, these carriers adeptly prolong the drug’s residence at injury locales. Their biodegradable and non-invasive nature ensures a harmonious interaction with the human physiology. These nano-carriers deftly modulate drug release within the gastrointestinal milieu, optimizing absorption and maximizing bioavailability. Beyond that, the strategic design of nanoparticles and microparticles ensures a precision-guided delivery to infection epicentres, adeptly curbing side effects and magnifying therapeutic efficacy.

Nanomaterial-based therapy is a promising tool against refractory bacterial infections, characterized by the ability to evade the existing mechanisms related to acquired drug resistance and increase the activity of antimicrobials (Makabenta et al., 2021). Figure 6 summarizes some nanotechnologies to overcome antibiotic resistance. Nano-form metals, metal oxides, and other nano-drugs also have direct antibacterial effects (Lee et al., 2019; Elbourne et al., 2020; Zhang et al., 2022).

Nanotechnology represents a promising platform for drug delivery, particularly in the fight against antibiotic resistance. The key advantages of these nanocarriers lie in their ability to optimize and enhance the therapeutic effects of drugs (Figure 6). Nanocarriers greatly improve the stability of antibiotics in complex physiological and pathological environments. For example, nano-metals and metal oxides offer a protective barrier against threats such as hydrolysis, oxidation, pH fluctuations, and enzymatic attacks, thereby ensuring the preservation of their activity until they reach the intended target site (Elbourne et al., 2020). Moreover, nanocarriers, including gels and polymer nanofibers, facilitate the sustained release of antibiotics due to their unique design (Thapa et al., 2020). This feature ensures consistent drug concentrations and helps reduce dosing frequencies, thereby enhancing patient compliance. Furthermore, their small size allows nanocarriers to reside longer at damaged or infected sites, enabling effective infection control precisely where it is most needed (Mofazzal Jahromi et al., 2018; Liang et al., 2022). Significantly, these nanocarriers possess biodegradable and non-invasive properties, allowing for their safe breakdown and elimination from the body after fulfilling their purpose without causing any further harm (Ohnstedt et al., 2019). Moreover, nanocarriers provide a protective environment for drugs in the gastrointestinal tract, regulating their release. For instance, combining or encapsulating polymyxins with polymers and liposomes can protect drugs in the gastrointestinal tract, leading to enhanced drug absorption (Maher et al., 2016; Faustino and Pinheiro, 2020). Furthermore, the design of nanos and microparticles ensures targeted drug delivery, enabling drugs to be directed straight to the infection site. This precision allows for more accurate treatments, reduced drug dosages, and diminished risks of systemic side effects (Lee et al., 2019; Zigrayova et al., 2023). Additionally, when combined with the most effective antibiotics, nano preparations have exhibited synergistic effects and the potential to address the emerging global crisis of bacterial resistance (Lee et al., 2019; Zigrayova et al., 2023). Chengyuan Qin et al. Overcoming colistin resistance in bacterial infections by negatively charged polyethylene glycol functionalized liposomal co-delivery of curcumin and colistin. Liposomes restored the affinity of mucilage to the bacterial membrane and increased the uptake of curcumin, thereby decreasing efflux pump activity and realizing the synergistic effect of mucilage and curcumin. The liposome-loaded group did not exhibit any toxicity at effective antibacterial doses (Qin et al., 2023).

## 6 The critical role and complexities of polymyxins in modern medicine

**FIGURE 7:**
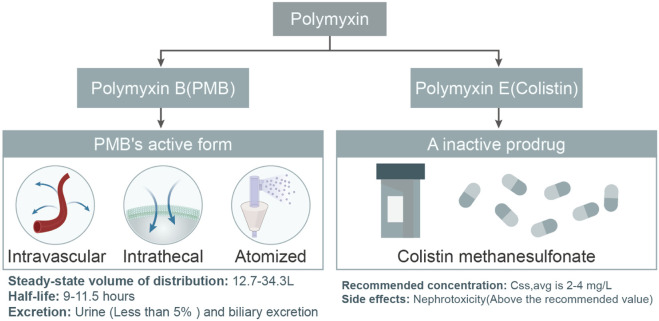
Comparative Pharmacokinetic Profiles of PMB and Colistin. This flowchart elucidates the distinct pharmacokinetic properties of PMB and Colistin. For PMB, the diagram underscores its distribution, half-life, and excretion pathways, notably the minimal renal excretion and a potential route through biliary excretion. In contrast, colistin is emphasized for its intravenous administration as the inactive prodrug, colistin methanesulphonate. The recommended concentrations for both drugs are depicted, with an accent on potential side effects, especially the heightened risk of nephrotoxicity when exceeding the advised levels.

Polymyxins, specifically Polymyxin B and Colistin (Polymyxin E), have emerged as pivotal agents in the fight against antibiotic-resistant bacterial infections. Polymyxin B is often available as a sulphate for injection, while colistin is available as polymyxin E sulphate for injection and colistin methane sulfonate for injection. CMS is a prodrug that is converted to colistin in the body after administration in order to exert antimicrobial activity. As the global medical community grapples with the challenges posed by multi-drug resistant Gram-negative bacteria (MDR-GNB), the significance and intricacies of these drugs have gained paramount importance. This article delves into the types, pharmacokinetic properties, differences, and the potential of personalized treatments with polymyxins. Additionally, it sheds light on the future research directions, emphasizing the need to further elucidate their behavior and interactions, especially in conjunction with contemporary medical treatments (Figure 7). CMS is a precursor drug that needs to be converted in the kidneys, and its conversion rate is affected by a number of factors, resulting in large individual variations in PK parameters, and blood concentrations are affected by renal function. PMB is mainly eliminated by non-renal routes and the total clearance has little correlation with renal function. Dosage does not need to be adjusted according to renal function, and effective blood mass concentrations can be achieved rapidly and are less affected by renal function. Therefore, PMB is more suitable for bloodstream infections, whereas CMS is more suitable for urinary tract infections. The antimicrobial activity of different polymyxin E formulations varies, with CMS being less active than polymyxin E sulphate and less toxic, while polymyxin E sulphate is less commonly used due to toxicity and is mainly used for drug sensitivity testing (Infectious Diseases Society of China et al., 2021). Currently, there are fewer data from studies related to polymyxin E sulphate, and it is expected that more relevant studies will enrich the clinical options in the future.

### 6.1 Types and drug forms of polymyxins

In current clinical practice, Polymyxin B and Colistin are widely regarded as the two primary polymyxins, both boasting impressive antibacterial properties. Due to the rising antibiotic resistance of MDR-GNB globally, many previously effective antibiotics have become less effective against MDR Gram-negative pathogens (El-Sayed Ahmed et al., 2020). This resurgence of colistin in the mid-1990s emerged as a crucial weapon against MDR Gram-negative pathogens (El-Sayed Ahmed et al., 2020). Notably, *Klebsiella pneumoniae*, a common cause of healthcare-associated infections, often uses colistin as the treatment of choice (Uzairue et al., 2022). However, these two polymyxins differ in their modes and forms of administration. Polymyxin E is commonly administered intravenously as the prodrug colistin methanesulphonate, while PMB can be directly intravenously administered as its active form under physiological pH (Nation et al., 2015) (Figure 7). Alarmingly, with the clinical discovery of more and more carbapenem resistant *pseudomonas*, especially resistance to polymyxins has begun to appear (Qureshi et al., 2015).

### 6.2 Pharmacokinetic properties of polymyxin B

The administration of PMB is versatile, including intravenous, intrathecal, or nebulized inhalation routes. After intravenous administration, its steady-state distribution volume ranges from 12.7–34.3 L, with a half-life of about 9–11.5 h. This provides valuable guidance for clinicians on dose adjustments. Interestingly, in both animals and humans, less than 5% of PMB is excreted in the urine, indicating that the kidneys are not the primary route of elimination for PMB (Zavascki et al., 2008; Manchandani et al., 2016). Although biliary excretion might contribute to PMB’s clearance, further studies are needed to identify other potential clearance mechanisms (Manchandani et al., 2016). It is worth noting that the intensity of concentration imposed by polymyxins affects the dynamics of genetic variation within the bacterial population, leading to different evolutionary outcomes of resistance. Jinxin Zhao et al. demonstrated that polymyxin B recurs at a critical threshold concentration (1 mg/L; i.e., 4× MIC) with low levels of resistance, but without fixed mutations, and that this resistance reverses upon removal of the antibiotic. This contrasts with the evolution of polymyxin B at ultra-MIC concentrations (≥4 × MIC), which drives the evolution of irreversible resistance, with molecular evolution occurring more rapidly at higher antibiotic concentrations. This study highlights the important role of combining evolutionary findings with pharmacokinetics/pharmacodynamics to optimize antibiotic use in patients (Zhao et al., 2022). V Aranzana-Climent et al. investigated a semi-mechanistic PK/PD model for the combination of polymyxin B and minocycline against polymyxin-resistant *Acinetobacter baumannii*. The combination effect was driven by minocycline, with PMB as an adjunct; simulations at clinically achievable concentrations indicated that 1.5 mg/L minocycline +0.2 mg/L PMB was expected to produce sustained killing for more than 30 h, whereas 0.3 mg/L minocycline +1 mg/L PMB was sufficient for bacterial regeneration. Interaction equations indicated that synergistic effects were maximized at PMB concentrations ≥0.1 mg/L and minocycline concentrations ≥1 mg/L. The possible mechanism is that PMB opens the bacterial membrane and increases the entry of minocycline into the cell, and the intracellular concentration of minocycline increased in bacteria treated with 0.5 mg/L PMB and minocycline in combination (Aranzana-Climent et al., 2020). Further studies on the protein binding rates of polymyxins and their combined antimicrobials in humans are necessary before any definitive recommendations can be issued. And, caution should be used in interpreting these modeling results based on *in vitro* results.

To provide the best treatment for each patient more accurately, researchers have begun to explore population pharmacokinetics (PPK). This method considers various physiological, pathological, and genetic factors that might influence drug efficacy and safety. Combined with pharmacodynamic (PD) metrics, this allows physicians to better adjust treatment strategies, achieving personalized treatment and enhancing therapeutic outcomes (van der Leeuw et al., 2014; Chen N. et al., 2022). Recent studies have assessed the pharmacokinetics/pharmacodynamics of Polymyxin B in patients with carbapenem-resistant *K. pneumoniae* bloodstream infections (Yu et al., 2022). Furthermore, for other drugs like paroxetine, population pharmacokinetic models have been successfully applied to guide personalized treatments (Li et al., 2022). These studies offer a framework for understanding how to leverage pharmacokinetic/pharmacodynamic principles to optimize polymyxin treatment strategies.

### 6.3 Metabolism, excretion of polymyxin B, and future research directions

While we have a certain understanding of many aspects of PMB, its metabolism and excretion mechanisms in the body remain somewhat elusive. Future research should focus more on these areas, especially considering the increasing bacterial resistance to conventional antibiotics. Additionally, with the advancements in medical technology, more patients now require continuous renal replacement therapy (CRRT) or extracorporeal membrane oxygenation (ECMO) support. These treatments might influence the pharmacokinetics of polymyxins, making it crucial to understand their interactions. A recent study reported a patient with septic shock induced by severe acute pancreatitis who received life support through ECMO and CRRT and multiple anti-infective drug treatments. The study monitored the plasma concentration of Colistin sulfate during ECMO and CRRT, finding no significant difference before and after ECMO and CRRT (Peng et al., 2022), implying that ECMO and CRRT might not significantly influence the pharmacokinetics of Colistin sulfate. Moreover, different connection modes for ECMO and CRRT have shown that both modalities can achieve therapeutic goals without necessitating higher levels of anticoagulation therapy (Liu et al., 2021), providing important guidance for clinicians on the use of polymyxins in patients receiving ECMO and CRRT treatments. In conclusion, as our understanding of the behavior of polymyxins in the body deepens, future research should focus on their interactions with modern medical technologies, such as ECMO and CRRT, to optimize treatment strategies and improve therapeutic outcomes.

## 7 Conclusion

This review provides an overview of the latest discoveries and development history of polymyxins, as well as the mechanisms underlying multidrug resistance to polymyxins. Additionally, we discuss current research directions to overcome polymyxin resistance and highlight new antibiotics undergoing clinical research. Furthermore, we outline the future challenges and prospects of polymyxins in treating bacterial infectious diseases, particularly in relation to MDR bacterial infections. Over the past 5 years, numerous studies have explored the impact of the *mcr* gene on polymyxin resistance, revealing a much more complex mechanism of bacterial drug resistance than previously understood. Future research should focus on elucidating the causes of colistin resistance to enable the precise design and development of antibiotic drugs targeting drug-resistant bacteria and optimize drug administration strategies. These efforts are crucial to minimize the development of resistance and prolong the effectiveness of polymyxin as a last-line treatment.

Different forms of polymyxins exhibit significant differences in pharmacokinetics and toxicity, with some forms being more prone to induce nephrotoxicity or neurotoxicity. This necessitates a deep evaluation and careful consideration by physicians when selecting and using polymyxins (Nation et al., 2014). Specifically, Polymyxin B is seen as the last line of defense against carbapenem-resistant microbes, but its common side effects, such as neurotoxicity and nephrotoxicity, cannot be overlooked (Nation and Li, 2009; Yu et al., 2022). The toxicity observed in clinical settings stems from colistin’s antibacterial mechanism, which involves the interaction and damage inflicted on bacterial bilayer membranes. When administered at high doses, this same mechanism can cause severe damage to the cell membranes of human organs, including the liver and kidneys. Toxicity is typically reversible upon discontinuation, and its severity is dose-dependent (Li et al., 2006). To address these issues, developing new polymyxin preparations must focus on reducing dosage, achieving targeted drug delivery, and controlling drug release. Using polymyxin-based nanoparticles, liposomes, microneedles, and composite nanomaterials necessitates collaborative efforts across disciplines such as chemistry, nanomedicine, and materials science to address polymyxin delivery, drug resistance, and toxicity challenges. Conjugation of polymyxin is also a promising approach that can enhance intestinal permeability and absorption while preventing microbial resistance. However, it is crucial to investigate the impact of molecular modifications on drug stability, antimicrobial activity, and the ability to overcome microbial drug resistance. The emergence of drug resistance poses a significant threat to the treatment of MDR-GNB infections since polymyxin serves as the last line of defense. Therefore, the future of antibiotic drug development lies in optimizing existing polymyxin drugs to reduce dosage, increase efficiency, mitigate toxicity, and overcome the emergence of drug resistance.

Addressing antibiotic resistance demands a comprehensive approach that includes political agendas, legislation, treatment development, and educational initiatives. Regular surveillance, policies, and the implementation of new medical therapies targeting resistant bacteria are essential for combating antibiotic resistance in human and agricultural contexts. Given the varying rates of resistance development across different antibiotics, multifaceted measures are necessary to ensure the sustainable development of healthcare. The increasing reliance on colistin as a last resort antimicrobial necessitates urgent exploration of its antimicrobial potential and the development of new, more effective antimicrobial agents to safeguard public health in the future. Looking ahead, there is growing interest in using new technological approaches to overcome polymyxin resistance. Continued advancements in analytical techniques for the identification and structural interpretation of natural products will likely lead to the discovery of novel polymyxin groups and new lipopeptide components within existing polymyxin groups.

## References

[B1] Abdallah M. Olafisoye O. Cortes C. Urban C. Landman D. Quale J. (2015). Activity of eravacycline against Enterobacteriaceae and Acinetobacter baumannii, including multidrug-resistant isolates, from New York City. Antimicrob. Agents Chemother. 59 (3), 1802–1805. 10.1128/AAC.04809-14 25534744 PMC4325809

[B2] Akova M. (2016). Epidemiology of antimicrobial resistance in bloodstream infections. Virulence 7 (3), 252–266. 10.1080/21505594.2016.1159366 26984779 PMC4871634

[B3] Aranzana-Climent V. Buyck J. M. Smani Y. Pachon-Diaz J. Marchand S. Couet W. (2020). Semi-mechanistic PK/PD modelling of combined polymyxin B and minocycline against a polymyxin-resistant strain of Acinetobacter baumannii. Clin. Microbiol. Infect. 26 (9), 1254 e1259–e1254. 10.1016/j.cmi.2020.01.017 32006693

[B4] Barbier F. Andremont A. Wolff M. Bouadma L. (2013). Hospital-acquired pneumonia and ventilator-associated pneumonia: recent advances in epidemiology and management. Curr. Opin. Pulm. Med. 19 (3), 216–228. 10.1097/MCP.0b013e32835f27be 23524477

[B5] Bassetti M. Righi E. Vena A. Graziano E. Russo A. Peghin M. (2018). Risk stratification and treatment of ICU-acquired pneumonia caused by multidrug-resistant/extensively drug-resistant/pandrug-resistant bacteria. Curr. Opin. Crit. Care 24 (5), 385–393. 10.1097/MCC.0000000000000534 30156569

[B6] Belaynehe K. M. Shin S. W. Park K. Y. Jang J. Y. Won H. G. Yoon I. J. (2018). Emergence of mcr-1 and mcr-3 variants coding for plasmid-mediated colistin resistance in *Escherichia coli* isolates from food-producing animals in South Korea. Int. J. Infect. Dis. 72, 22–24. 10.1016/j.ijid.2018.05.011 29803875

[B7] Benedict R. G. Langlykke A. F. (1947). Antibiotic activity of Bacillus polymyxa. J. Bacteriol. 54 (1), 24. 20344246

[B8] Bengoechea J. A. Skurnik M. (2000). Temperature-regulated efflux pump/potassium antiporter system mediates resistance to cationic antimicrobial peptides in Yersinia. Mol. Microbiol. 37 (1), 67–80. 10.1046/j.1365-2958.2000.01956.x 10931306

[B9] Bialvaei A. Z. Samadi Kafil H. (2015). Colistin, mechanisms and prevalence of resistance. Curr. Med. Res. Opin. 31 (4), 707–721. 10.1185/03007995.2015.1018989 25697677

[B10] Bojkovic J. Richie D. L. Six D. A. Rath C. M. Sawyer W. S. Hu Q. (2015). Characterization of an acinetobacter baumannii lptD deletion strain: permeability defects and response to inhibition of lipopolysaccharide and fatty acid biosynthesis. J. Bacteriol. 198 (4), 731–741. 10.1128/JB.00639-15 26668262 PMC4751815

[B11] Brownlee G. Bushby S. R. (1948). Chemotherapy and pharmacology of aerosporin; a selective gram-negative antibiotic. Lancet 1 (6491), 127–132. 10.1016/s0140-6736(48)90090-7 18920131

[B12] Cai Y. Chai D. Wang R. Liang B. Bai N. (2012). Colistin resistance of Acinetobacter baumannii: clinical reports, mechanisms and antimicrobial strategies. J. Antimicrob. Chemother. 67 (7), 1607–1615. 10.1093/jac/dks084 22441575

[B13] Cannatelli A. Giani T. D'Andrea M. M. Di Pilato V. Arena F. Conte V. (2014). MgrB inactivation is a common mechanism of colistin resistance in KPC-producing *Klebsiella pneumoniae* of clinical origin. Antimicrob. Agents Chemother. 58 (10), 5696–5703. 10.1128/AAC.03110-14 25022583 PMC4187966

[B14] Carfrae L. A. Rachwalski K. French S. Gordzevich R. Seidel L. Tsai C. N. (2023). Inhibiting fatty acid synthesis overcomes colistin resistance. Nat. Microbiol. 8 (6), 1026–1038. 10.1038/s41564-023-01369-z 37127701

[B15] Cheah S. E. Johnson M. D. Zhu Y. Tsuji B. T. Forrest A. Bulitta J. B. (2016). Polymyxin resistance in acinetobacter baumannii: genetic mutations and transcriptomic changes in response to clinically relevant dosage regimens. Sci. Rep. 6, 26233. 10.1038/srep26233 27195897 PMC4872528

[B16] Chen J. Lu Y. Ye F. Zhang H. Zhou Y. Li J. (2022a). A small-molecule inhibitor of the anthranilyl-CoA Synthetase PqsA for the treatment of multidrug-resistant *Pseudomonas aeruginosa* . Microbiol. Spectr. 10 (4), e0276421. 10.1128/spectrum.02764-21 35856709 PMC9430567

[B17] Chen N. Guo J. Xie J. Xu M. Hao X. Ma K. (2022b). Population pharmacokinetics of polymyxin B: a systematic review. Ann. Transl. Med. 10 (4), 231. 10.21037/atm-22-236 35280373 PMC8908148

[B18] Deris Z. Z. Akter J. Sivanesan S. Roberts K. D. Thompson P. E. Nation R. L. (2014). A secondary mode of action of polymyxins against Gram-negative bacteria involves the inhibition of NADH-quinone oxidoreductase activity. J. Antibiot. (Tokyo) 67 (2), 147–151. 10.1038/ja.2013.111 24169795 PMC3943757

[B19] Dixon R. A. Chopra I. (1986). Leakage of periplasmic proteins from *Escherichia coli* mediated by polymyxin B nonapeptide. Antimicrob. Agents Chemother. 29 (5), 781–788. 10.1128/AAC.29.5.781 3015004 PMC284154

[B20] Doi Y. Murray G. L. Peleg A. Y. (2015). Acinetobacter baumannii: evolution of antimicrobial resistance-treatment options. Semin. Respir. Crit. Care Med. 36 (1), 85–98. 10.1055/s-0034-1398388 25643273 PMC4465586

[B21] Doymaz M. Z. Karaaslan E. (2019). Comparison of antibacterial activities of polymyxin B and colistin against multidrug resistant Gram negative bacteria. Infect. Dis. (Lond) 51 (9), 676–682. 10.1080/23744235.2019.1640386 31298061

[B22] Dwyer D. J. Belenky P. A. Yang J. H. MacDonald I. C. Martell J. D. Takahashi N. (2014). Antibiotics induce redox-related physiological alterations as part of their lethality. Proc. Natl. Acad. Sci. U. S. A. 111 (20), E2100–E2109. 10.1073/pnas.1401876111 24803433 PMC4034191

[B23] Elbourne A. Cheeseman S. Atkin P. Truong N. P. Syed N. Zavabeti A. (2020). Antibacterial liquid metals: biofilm treatment via magnetic activation. ACS Nano 14 (1), 802–817. 10.1021/acsnano.9b07861 31922722

[B24] El-Sayed Ahmed M. A. E. Zhong L. L. Shen C. Yang Y. Doi Y. Tian G. B. (2020). Colistin and its role in the Era of antibiotic resistance: an extended review (2000-2019). Emerg. Microbes Infect. 9 (1), 868–885. 10.1080/22221751.2020.1754133 32284036 PMC7241451

[B25] Falagas M. E. Kasiakou S. K. (2005). Colistin: the revival of polymyxins for the management of multidrug-resistant gram-negative bacterial infections. Clin. Infect. Dis. 40 (9), 1333–1341. 10.1086/429323 15825037

[B26] Falagas M. E. Rafailidis P. I. Matthaiou D. K. (2010). Resistance to polymyxins: mechanisms, frequency and treatment options. Drug Resist Updat 13 (4-5), 132–138. 10.1016/j.drup.2010.05.002 20843473

[B27] Faustino C. Pinheiro L. (2020). Lipid systems for the delivery of amphotericin B in antifungal therapy. Pharmaceutics 12 (1), 29. 10.3390/pharmaceutics12010029 31906268 PMC7023008

[B28] Fernandez L. Gooderham W. J. Bains M. McPhee J. B. Wiegand I. Hancock R. E. (2010). Adaptive resistance to the "last hope" antibiotics polymyxin B and colistin in *Pseudomonas aeruginosa* is mediated by the novel two-component regulatory system ParR-ParS. Antimicrob. Agents Chemother. 54 (8), 3372–3382. 10.1128/AAC.00242-10 20547815 PMC2916309

[B29] Giddins M. J. Macesic N. Annavajhala M. K. Stump S. Khan S. McConville T. H. (2018). Successive emergence of ceftazidime-avibactam resistance through distinct genomic adaptations in bla(KPC-2)-Harboring *Klebsiella pneumoniae* sequence type 307 isolates. Antimicrob. Agents Chemother. 62 (3), e02101. 10.1128/AAC.02101-17 29263067 PMC5826117

[B30] Gilleland H. E. Jr. Farley L. B. (1982). Adaptive resistance to polymyxin in *Pseudomonas aeruginosa* due to an outer membrane impermeability mechanism. Can. J. Microbiol. 28 (7), 830–840. 10.1139/m82-125 6293694

[B31] Gilleland H. E. Jr. Lyle R. D. (1979). Chemical alterations in cell envelopes of polymyxin-resistant *Pseudomonas aeruginosa* isolates. J. Bacteriol. 138 (3), 839–845. 10.1128/jb.138.3.839-845.1979 222726 PMC218112

[B32] Gonzalez-Avila L. U. Loyola-Cruz M. A. Hernandez-Cortez C. Bello-Lopez J. M. Castro-Escarpulli G. (2021). Colistin resistance in aeromonas spp. Int. J. Mol. Sci. 22 (11), 5974. 10.3390/ijms22115974 34205867 PMC8199210

[B33] Govaerts C. Orwa J. Van Schepdael A. Roets E. Hoogmartens J. (2002). Characterization of polypeptide antibiotics of the polymyxin series by liquid chromatography electrospray ionization ion trap tandem mass spectrometry. J. Pept. Sci. 8 (2), 45–55. 10.1002/psc.367 11860028

[B34] Groisman E. A. Kayser J. Soncini F. C. (1997). Regulation of polymyxin resistance and adaptation to low-Mg2+ environments. J. Bacteriol. 179 (22), 7040–7045. 10.1128/jb.179.22.7040-7045.1997 9371451 PMC179645

[B35] Gutu A. D. Sgambati N. Strasbourger P. Brannon M. K. Jacobs M. A. Haugen E. (2013). Polymyxin resistance of *Pseudomonas aeruginosa* phoQ mutants is dependent on additional two-component regulatory systems. Antimicrob. Agents Chemother. 57 (5), 2204–2215. 10.1128/AAC.02353-12 23459479 PMC3632916

[B36] Hancock R. E. (1997). The bacterial outer membrane as a drug barrier. Trends Microbiol. 5 (1), 37–42. 10.1016/S0966-842X(97)81773-8 9025234

[B37] Hood M. I. Becker K. W. Roux C. M. Dunman P. M. Skaar E. P. (2013). Genetic determinants of intrinsic colistin tolerance in Acinetobacter baumannii. Infect. Immun. 81 (2), 542–551. 10.1128/IAI.00704-12 23230287 PMC3553813

[B38] Infectious Diseases Society of China Chinese Thoracic Society Chinese Society of Critical Care Medicine Chinese Society of Hematology Chinese Society of Bacterial Infection and Resistance Committee of Drug Clinical Evaluate Research of Chinese Pharmaceutical Association (2021). Multi-disciplinary expert consensus on the optimal clinical use of the polymyxins in China. Zhonghua Jie He He Hu Xi Za Zhi 44 (4), 292–310. 10.3760/cma.j.cn112147-20201109-01091 33832018

[B39] Jayol A. Nordmann P. Brink A. Poirel L. (2015a). Heteroresistance to colistin in *Klebsiella pneumoniae* associated with alterations in the PhoPQ regulatory system. Antimicrob. Agents Chemother. 59 (5), 2780–2784. 10.1128/AAC.05055-14 25733503 PMC4394806

[B40] Jayol A. Nordmann P. Desroches M. Decousser J. W. Poirel L. (2016). Acquisition of broad-spectrum cephalosporin resistance leading to colistin resistance in *Klebsiella pneumoniae* . Antimicrob. Agents Chemother. 60 (5), 3199–3201. 10.1128/AAC.00237-16 26953194 PMC4862518

[B41] Jayol A. Poirel L. Villegas M. V. Nordmann P. (2015b). Modulation of mgrB gene expression as a source of colistin resistance in Klebsiella oxytoca. Int. J. Antimicrob. Agents 46 (1), 108–110. 10.1016/j.ijantimicag.2015.02.015 25982250

[B42] Jeannot K. Bolard A. Plesiat P. (2017). Resistance to polymyxins in Gram-negative organisms. Int. J. Antimicrob. Agents 49 (5), 526–535. 10.1016/j.ijantimicag.2016.11.029 28163137

[B43] Jiang S. S. Liu M. C. Teng L. J. Wang W. B. Hsueh P. R. Liaw S. J. (2010). *Proteus mirabilis* pmrI, an RppA-regulated gene necessary for polymyxin B resistance, biofilm formation, and urothelial cell invasion. Antimicrob. Agents Chemother. 54 (4), 1564–1571. 10.1128/AAC.01219-09 20123999 PMC2849355

[B44] Kohanski M. A. Dwyer D. J. Hayete B. Lawrence C. A. Collins J. J. (2007). A common mechanism of cellular death induced by bactericidal antibiotics. Cell 130 (5), 797–810. 10.1016/j.cell.2007.06.049 17803904

[B45] Kwa A. Kasiakou S. K. Tam V. H. Falagas M. E. (2007). Polymyxin B: similarities to and differences from colistin (polymyxin E). Expert Rev. Anti Infect. Ther. 5 (5), 811–821. 10.1586/14787210.5.5.811 17914915

[B46] Lee N. Y. Ko W. C. Hsueh P. R. (2019). Nanoparticles in the treatment of infections caused by multidrug-resistant organisms. Front. Pharmacol. 10, 1153. 10.3389/fphar.2019.01153 31636564 PMC6787836

[B47] Li J. Nation R. L. Turnidge J. D. Milne R. W. Coulthard K. Rayner C. R. (2006). Colistin: the re-emerging antibiotic for multidrug-resistant Gram-negative bacterial infections. Lancet Infect. Dis. 6 (9), 589–601. 10.1016/S1473-3099(06)70580-1 16931410

[B48] Li J. Zhang X. Han N. Wan P. Zhao F. Xu T. (2023). Mechanism of action of isopropoxy benzene guanidine against multidrug-resistant pathogens. Microbiol. Spectr. 11 (1), e0346922. 10.1128/spectrum.03469-22 36475769 PMC9927234

[B49] Li X. L. Huang S. Q. Xiao T. Wang X. P. Kong W. Liu S. J. (2022). Pharmacokinetics of immediate and sustained-release formulations of paroxetine: population pharmacokinetic approach to guide paroxetine personalized therapy in Chinese psychotic patients. Front. Pharmacol. 13, 966622. 10.3389/fphar.2022.966622 36172189 PMC9510632

[B50] Liang Y. Liang Y. Zhang H. Guo B. (2022). Antibacterial biomaterials for skin wound dressing. Asian J. Pharm. Sci. 17 (3), 353–384. 10.1016/j.ajps.2022.01.001 35782328 PMC9237601

[B51] Lima W. G. Alves M. C. Cruz W. S. Paiva M. C. (2018). Chromosomally encoded and plasmid-mediated polymyxins resistance in Acinetobacter baumannii: a huge public health threat. Eur. J. Clin. Microbiol. Infect. Dis. 37 (6), 1009–1019. 10.1007/s10096-018-3223-9 29524060

[B52] Lin H. Liu X. Sun P. (2022). Effects of aerosol inhalation combined with intravenous drip of polymyxin B on bacterial clearance, symptoms improvement, and serum infection indexes in patients with pneumonia induced by multidrug-resistant gram-negative bacteria. Emerg. Med. Int. 2022, 5244538. 10.1155/2022/5244538 36072613 PMC9441374

[B53] Lin Q. Y. Tsai Y. L. Liu M. C. Lin W. C. Hsueh P. R. Liaw S. J. (2014). *Serratia marcescens* arn, a PhoP-regulated locus necessary for polymyxin B resistance. Antimicrob. Agents Chemother. 58 (9), 5181–5190. 10.1128/AAC.00013-14 24957827 PMC4135846

[B54] Liu J. H. Liu Y. Y. Shen Y. B. Yang J. Walsh T. R. Wang Y. (2024). Plasmid-mediated colistin-resistance genes: mcr. Trends Microbiol. 32 (4), 365–378. 10.1016/j.tim.2023.10.006 38008597

[B55] Liu M. Yan Y. Li G. Zhang Y. Guo F. (2021). Comparison of clinical outcomes of different connection modes of extracorporeal membrane oxygenation combine with continuous renal replacement therapy. Heart Surg. Forum 24 (6), E1018–E1022. 10.1532/hsf.4335 34962461

[B56] Liu Y. Jia Y. Yang K. Tong Z. Shi J. Li R. (2020). Melatonin overcomes MCR-mediated colistin resistance in Gram-negative pathogens. Theranostics 10 (23), 10697–10711. 10.7150/thno.45951 32929375 PMC7482817

[B57] Liu Y. Y. Wang Y. Walsh T. R. Yi L. X. Zhang R. Spencer J. (2016). Emergence of plasmid-mediated colistin resistance mechanism MCR-1 in animals and human beings in China: a microbiological and molecular biological study. Lancet Infect. Dis. 16 (2), 161–168. 10.1016/S1473-3099(15)00424-7 26603172

[B58] MacNair C. R. Stokes J. M. Carfrae L. A. Fiebig-Comyn A. A. Coombes B. K. Mulvey M. R. (2018). Overcoming mcr-1 mediated colistin resistance with colistin in combination with other antibiotics. Nat. Commun. 9 (1), 458. 10.1038/s41467-018-02875-z 29386620 PMC5792607

[B59] Maher S. Mrsny R. J. Brayden D. J. (2016). Intestinal permeation enhancers for oral peptide delivery. Adv. Drug Deliv. Rev. 106 (Pt B), 277–319. 10.1016/j.addr.2016.06.005 27320643

[B60] Makabenta J. M. V. Nabawy A. Li C. H. Schmidt-Malan S. Patel R. Rotello V. M. (2021). Nanomaterial-based therapeutics for antibiotic-resistant bacterial infections. Nat. Rev. Microbiol. 19 (1), 23–36. 10.1038/s41579-020-0420-1 32814862 PMC8559572

[B61] Manchandani P. Zhou J. Ledesma K. R. Truong L. D. Chow D. S. Eriksen J. L. (2016). Characterization of polymyxin B biodistribution and disposition in an animal model. Antimicrob. Agents Chemother. 60 (2), 1029–1034. 10.1128/AAC.02445-15 26643340 PMC4750689

[B62] Mancuso G. Midiri A. Gerace E. Biondo C. (2021). Bacterial antibiotic resistance: the most critical pathogens. Pathogens 10 (10), 1310. 10.3390/pathogens10101310 34684258 PMC8541462

[B63] McLeod G. I. Spector M. P. (1996). Starvation- and Stationary-phase-induced resistance to the antimicrobial peptide polymyxin B in *Salmonella typhimurium* is RpoS (sigma(S)) independent and occurs through both phoP-dependent and -independent pathways. J. Bacteriol. 178 (13), 3683–3688. 10.1128/jb.178.13.3683-3688.1996 8682766 PMC178147

[B64] Miethke M. Pieroni M. Weber T. Bronstrup M. Hammann P. Halby L. (2021). Towards the sustainable discovery and development of new antibiotics. Nat. Rev. Chem. 5 (10), 726–749. 10.1038/s41570-021-00313-1 PMC8374425 34426795

[B65] Mlynarcik P. Kolar M. (2019). Molecular mechanisms of polymyxin resistance and detection of mcr genes. Biomed. Pap. Med. Fac. Univ. Palacky. Olomouc Czech Repub. 163 (1), 28–38. 10.5507/bp.2018.070 30439931

[B66] Mofazzal Jahromi M. A. Sahandi Zangabad P. Moosavi Basri S. M. Sahandi Zangabad K. Ghamarypour A. Aref A. R. (2018). Nanomedicine and advanced technologies for burns: preventing infection and facilitating wound healing. Adv. Drug Deliv. Rev. 123, 33–64. 10.1016/j.addr.2017.08.001 28782570 PMC5742034

[B67] Moffatt J. H. Harper M. Boyce J. D. (2019). Mechanisms of polymyxin resistance. Adv. Exp. Med. Biol. 1145, 55–71. 10.1007/978-3-030-16373-0_5 31364071

[B68] Moffatt J. H. Harper M. Harrison P. Hale J. D. Vinogradov E. Seemann T. (2010). Colistin resistance in Acinetobacter baumannii is mediated by complete loss of lipopolysaccharide production. Antimicrob. Agents Chemother. 54 (12), 4971–4977. 10.1128/AAC.00834-10 20855724 PMC2981238

[B69] Mogi T. Murase Y. Mori M. Shiomi K. Omura S. Paranagama M. P. (2009). Polymyxin B identified as an inhibitor of alternative NADH dehydrogenase and malate: quinone oxidoreductase from the Gram-positive bacterium Mycobacterium smegmatis. J. Biochem. 146 (4), 491–499. 10.1093/jb/mvp096 19564154

[B70] Moore R. A. Chan L. Hancock R. E. (1984). Evidence for two distinct mechanisms of resistance to polymyxin B in *Pseudomonas aeruginosa* . Antimicrob. Agents Chemother. 26 (4), 539–545. 10.1128/AAC.26.4.539 6097168 PMC179960

[B71] Morey P. Viadas C. Euba B. Hood D. W. Barberan M. Gil C. (2013). Relative contributions of lipooligosaccharide inner and outer core modifications to nontypeable Haemophilus influenzae pathogenesis. Infect. Immun. 81 (11), 4100–4111. 10.1128/IAI.00492-13 23980106 PMC3811809

[B72] Morrissey I. Olesky M. Hawser S. Lob S. H. Karlowsky J. A. Corey G. R. (2020). *In vitro* activity of eravacycline against gram-negative bacilli isolated in clinical Laboratories worldwide from 2013 to 2017. Antimicrob. Agents Chemother. 64 (3), e01699. 10.1128/AAC.01699-19 31843999 PMC7038303

[B73] Nadimpalli M. L. Marks S. J. Montealegre M. C. Gilman R. H. Pajuelo M. J. Saito M. (2020). Urban informal settlements as hotspots of antimicrobial resistance and the need to curb environmental transmission. Nat. Microbiol. 5 (6), 787–795. 10.1038/s41564-020-0722-0 32467623

[B74] Nang S. C. Azad M. A. K. Velkov T. Zhou Q. T. Li J. (2021). Rescuing the last-line polymyxins: achievements and challenges. Pharmacol. Rev. 73 (2), 679–728. 10.1124/pharmrev.120.000020 33627412 PMC7911091

[B75] Nation R. L. Li J. (2009). Colistin in the 21st century. Curr. Opin. Infect. Dis. 22 (6), 535–543. 10.1097/QCO.0b013e328332e672 19797945 PMC2869076

[B76] Nation R. L. Li J. Cars O. Couet W. Dudley M. N. Kaye K. S. (2015). Framework for optimisation of the clinical use of colistin and polymyxin B: the Prato polymyxin consensus. Lancet Infect. Dis. 15 (2), 225–234. 10.1016/S1473-3099(14)70850-3 25459221

[B77] Nation R. L. Velkov T. Li J. (2014). Colistin and polymyxin B: peas in a pod, or chalk and cheese? Clin. Infect. Dis. 59 (1), 88–94. 10.1093/cid/ciu213 24700659 PMC4305129

[B78] Needham B. D. Trent M. S. (2013). Fortifying the barrier: the impact of lipid A remodelling on bacterial pathogenesis. Nat. Rev. Microbiol. 11 (7), 467–481. 10.1038/nrmicro3047 23748343 PMC6913092

[B79] Nikaido H. (2003). Molecular basis of bacterial outer membrane permeability revisited. Microbiol. Mol. Biol. Rev. 67 (4), 593–656. 10.1128/MMBR.67.4.593-656.2003 14665678 PMC309051

[B80] Niu B. Vater J. Rueckert C. Blom J. Lehmann M. Ru J. J. (2013). Polymyxin P is the active principle in suppressing phytopathogenic Erwinia spp. by the biocontrol rhizobacterium Paenibacillus polymyxa M-1. BMC Microbiol. 13, 137. 10.1186/1471-2180-13-137 23773687 PMC3708778

[B81] Nordmann P. Jayol A. Poirel L. (2016). Rapid detection of polymyxin resistance in enterobacteriaceae. Emerg. Infect. Dis. 22 (6), 1038–1043. 10.3201/eid2206.151840 27191712 PMC4880072

[B82] Ohnstedt E. Lofton Tomenius H. Vagesjo E. Phillipson M. (2019). The discovery and development of topical medicines for wound healing. Expert Opin. Drug Discov. 14 (5), 485–497. 10.1080/17460441.2019.1588879 30870037

[B83] Olaitan A. O. Morand S. Rolain J. M. (2014). Mechanisms of polymyxin resistance: acquired and intrinsic resistance in bacteria. Front. Microbiol. 5, 643. 10.3389/fmicb.2014.00643 25505462 PMC4244539

[B84] Pelletier M. R. Casella L. G. Jones J. W. Adams M. D. Zurawski D. V. Hazlett K. R. (2013). Unique structural modifications are present in the lipopolysaccharide from colistin-resistant strains of Acinetobacter baumannii. Antimicrob. Agents Chemother. 57 (10), 4831–4840. 10.1128/AAC.00865-13 23877686 PMC3811424

[B85] Peng D. Zhang F. Lv P. Chen Y. Yang J. Zhu W. (2022). Plasma concentrations of Colistin sulfate in a patient with septic shock on extracorporeal membrane oxygenation and continuous renal replacement therapy: a case report. Ann. Transl. Med. 10 (10), 614. 10.21037/atm-22-2081 35722356 PMC9201130

[B86] Poirel L. Jayol A. Nordmann P. (2017). Polymyxins: antibacterial activity, susceptibility testing, and resistance mechanisms encoded by plasmids or chromosomes. Clin. Microbiol. Rev. 30 (2), 557–596. 10.1128/CMR.00064-16 28275006 PMC5355641

[B87] Pulingam T. Parumasivam T. Gazzali A. M. Sulaiman A. M. Chee J. Y. Lakshmanan M. (2022). Antimicrobial resistance: prevalence, economic burden, mechanisms of resistance and strategies to overcome. Eur. J. Pharm. Sci. 170, 106103. 10.1016/j.ejps.2021.106103 34936936

[B88] Qin C. Tang N. Gan Y. Zhao H. Li Y. Tian G. B. (2023). Liposomes Co-delivering curcumin and colistin to overcome colistin resistance in bacterial infections. Adv. Healthc. Mater 12 (24), e2202903. 10.1002/adhm.202202903 37523195

[B89] Qureshi Z. A. Hittle L. E. O'Hara J. A. Rivera J. I. Syed A. Shields R. K. (2015). Colistin-resistant Acinetobacter baumannii: beyond carbapenem resistance. Clin. Infect. Dis. 60 (9), 1295–1303. 10.1093/cid/civ048 25632010 PMC4462660

[B90] Roberts K. D. Zhu Y. Azad M. A. K. Han M. L. Wang J. Wang L. (2022). A synthetic lipopeptide targeting top-priority multidrug-resistant Gram-negative pathogens. Nat. Commun. 13 (1), 1625. 10.1038/s41467-022-29234-3 35338128 PMC8956739

[B91] Rodriguez-Santiago J. Cornejo-Juarez P. Silva-Sanchez J. Garza-Ramos U. (2021). Polymyxin resistance in Enterobacterales: overview and epidemiology in the Americas. Int. J. Antimicrob. Agents 58 (5), 106426. 10.1016/j.ijantimicag.2021.106426 34419579

[B92] Rutten A. A. Bequet-Passelecq B. G. Koeter H. B. (1990). Two-compartment model for rabbit skin organ culture. Vitro Cell Dev. Biol. 26 (4), 353–360. 10.1007/BF02623826 1693141

[B93] Sampson T. R. Liu X. Schroeder M. R. Kraft C. S. Burd E. M. Weiss D. S. (2012). Rapid killing of Acinetobacter baumannii by polymyxins is mediated by a hydroxyl radical death pathway. Antimicrob. Agents Chemother. 56 (11), 5642–5649. 10.1128/AAC.00756-12 22908157 PMC3486575

[B94] Sato T. Shiraishi T. Hiyama Y. Honda H. Shinagawa M. Usui M. (2018). Contribution of novel amino acid alterations in PmrA or PmrB to colistin resistance in mcr-negative *Escherichia coli* clinical isolates, including major multidrug-resistant lineages O25b:H4-st131-H30Rx and non-x. Antimicrob. Agents Chemother. 62 (9), e00864. 10.1128/AAC.00864-18 29914952 PMC6125499

[B95] Schindler M. Osborn M. J. (1979). Interaction of divalent cations and polymyxin B with lipopolysaccharide. Biochemistry 18 (20), 4425–4430. 10.1021/bi00587a024 226126

[B96] Schrader S. M. Vaubourgeix J. Nathan C. (2020). Biology of antimicrobial resistance and approaches to combat it. Sci. Transl. Med. 12 (549), eaaz6992. 10.1126/scitranslmed.aaz6992 32581135 PMC8177555

[B97] Schromm A. B. Paulowski L. Kaconis Y. Kopp F. Koistinen M. Donoghue A. (2021). Cathelicidin and PMB neutralize endotoxins by multifactorial mechanisms including LPS interaction and targeting of host cell membranes. Proc. Natl. Acad. Sci. U. S. A. 118 (27), e2101721118. 10.1073/pnas.2101721118 34183393 PMC8271772

[B98] Schwarz S. Johnson A. P. (2016). Transferable resistance to colistin: a new but old threat. J. Antimicrob. Chemother. 71 (8), 2066–2070. 10.1093/jac/dkw274 27342545

[B99] Shein A. M. S. Wannigama D. L. Higgins P. G. Hurst C. Abe S. Hongsing P. (2022). High prevalence of mgrB-mediated colistin resistance among carbapenem-resistant *Klebsiella pneumoniae* is associated with biofilm formation, and can be overcome by colistin-EDTA combination therapy. Sci. Rep. 12 (1), 12939. 10.1038/s41598-022-17083-5 35902639 PMC9334626

[B100] Sherry N. Howden B. (2018). Emerging Gram negative resistance to last-line antimicrobial agents fosfomycin, colistin and ceftazidime-avibactam - epidemiology, laboratory detection and treatment implications. Expert Rev. Anti Infect. Ther. 16 (4), 289–306. 10.1080/14787210.2018.1453807 29543500

[B101] Shoji J. Kato T. Hinoo H. (1977). The structures of two new polymyxin group antibiotics. J. Antibiot. (Tokyo) 30 (5), 427–429. 10.7164/antibiotics.30.427 195923

[B102] Snitkin E. S. Zelazny A. M. Gupta J. Program N. C. S. Palmore T. N. Murray P. R. (2013). Genomic insights into the fate of colistin resistance and Acinetobacter baumannii during patient treatment. Genome Res. 23 (7), 1155–1162. 10.1101/gr.154328.112 23564252 PMC3698508

[B103] Srisakul S. Wannigama D. L. Higgins P. G. Hurst C. Abe S. Hongsing P. (2022). Overcoming addition of phosphoethanolamine to lipid A mediated colistin resistance in Acinetobacter baumannii clinical isolates with colistin-sulbactam combination therapy. Sci. Rep. 12 (1), 11390. 10.1038/s41598-022-15386-1 35794134 PMC9259700

[B104] Stansly P. G. Brownlee G. (1949). Nomenclature of polymyxin antibiotics. Nature 163 (4146), 611. 10.1038/163611a0 18117140

[B105] Stokes J. M. MacNair C. R. Ilyas B. French S. Cote J. P. Bouwman C. (2017). Pentamidine sensitizes Gram-negative pathogens to antibiotics and overcomes acquired colistin resistance. Nat. Microbiol. 2, 17028. 10.1038/nmicrobiol.2017.28 28263303 PMC5360458

[B106] Storm D. R. Rosenthal K. S. Swanson P. E. (1977). Polymyxin and related peptide antibiotics. Annu. Rev. Biochem. 46, 723–763. 10.1146/annurev.bi.46.070177.003451 197881

[B107] Szucs Z. Bereczki I. Fenyvesi F. Herczegh P. Ostorhazi E. Borbas A. (2022). Synthesis of an amphiphilic vancomycin aglycone derivative inspired by polymyxins: overcoming glycopeptide resistance in Gram-positive and Gram-negative bacteria in synergy with teicoplanin *in vitro* . Sci. Rep. 12 (1), 20921. 10.1038/s41598-022-24807-0 36463278 PMC9719540

[B108] Tang T. Li Y. Xu P. Zhong Y. Yang M. Ma W. (2023). Optimization of polymyxin B regimens for the treatment of carbapenem-resistant organism nosocomial pneumonia: a real-world prospective study. Crit. Care 27 (1), 164. 10.1186/s13054-023-04448-z 37106370 PMC10142183

[B109] Thapa R. K. Diep D. B. Tonnesen H. H. (2020). Topical antimicrobial peptide formulations for wound healing: current developments and future prospects. Acta Biomater. 103, 52–67. 10.1016/j.actbio.2019.12.025 31874224

[B110] Thi Khanh Nhu N. Riordan D. W. Do Hoang Nhu T. Thanh D. P. Thwaites G. Huong Lan N. P. (2016). The induction and identification of novel Colistin resistance mutations in Acinetobacter baumannii and their implications. Sci. Rep. 6, 28291. 10.1038/srep28291 27329501 PMC4916428

[B111] Tomczyk S. Zanichelli V. Grayson M. L. Twyman A. Abbas M. Pires D. (2019). Control of carbapenem-resistant enterobacteriaceae, acinetobacter baumannii, and *Pseudomonas aeruginosa* in healthcare facilities: a systematic review and reanalysis of quasi-experimental studies. Clin. Infect. Dis. 68 (5), 873–884. 10.1093/cid/ciy752 30475989 PMC6389314

[B112] Tzeng Y. L. Ambrose K. D. Zughaier S. Zhou X. Miller Y. K. Shafer W. M. (2005). Cationic antimicrobial peptide resistance in Neisseria meningitidis. J. Bacteriol. 187 (15), 5387–5396. 10.1128/JB.187.15.5387-5396.2005 16030233 PMC1196002

[B113] Tzeng Y. L. Berman Z. Toh E. Bazan J. A. Turner A. N. Retchless A. C. (2019). Heteroresistance to the model antimicrobial peptide polymyxin B in the emerging Neisseria meningitidis lineage 11.2 urethritis clade: mutations in the pilMNOPQ operon. Mol. Microbiol. 111 (1), 254–268. 10.1111/mmi.14153 30338585 PMC6351203

[B114] Uzairue L. I. Rabaan A. A. Adewumi F. A. Okolie O. J. Folorunso J. B. Bakhrebah M. A. (2022). Global prevalence of colistin resistance in *Klebsiella pneumoniae* from bloodstream infection: a systematic review and meta-analysis. Pathogens 11 (10), 1092. 10.3390/pathogens11101092 36297149 PMC9607870

[B115] van der Leeuw J. Ridker P. M. van der Graaf Y. Visseren F. L. (2014). Personalized cardiovascular disease prevention by applying individualized prediction of treatment effects. Eur. Heart J. 35 (13), 837–843. 10.1093/eurheartj/ehu004 24513790

[B116] Velkov T. Thompson P. E. Nation R. L. Li J. (2010). Structure--activity relationships of polymyxin antibiotics. J. Med. Chem. 53 (5), 1898–1916. 10.1021/jm900999h 19874036 PMC2907661

[B117] Wang L. Zhang Y. Lin Y. Cao J. Xu C. Chen L. (2023). Resveratrol increases sensitivity of clinical colistin-resistant *Pseudomonas aeruginosa* to colistin *in vitro* and *in vivo* . Microbiol. Spectr. 11 (1), e0199222. 10.1128/spectrum.01992-22 36475724 PMC9927286

[B118] Whitfield C. Trent M. S. (2014). Biosynthesis and export of bacterial lipopolysaccharides. Annu. Rev. Biochem. 83, 99–128. 10.1146/annurev-biochem-060713-035600 24580642

[B119] Yang X. Guo C. Wu G. Zhao K. Xiang D. Xu D. (2023). Treatment of central nervous system infection caused by multidrug-resistant acinetobacter baumannii with intravenous and intraventricular colistin sulfate: a case report and literature review. Infect. Drug Resist 16, 6029–6038. 10.2147/IDR.S425415 37719653 PMC10503561

[B120] Yap P. S. Cheng W. H. Chang S. K. Lim S. E. Lai K. S. (2022). MgrB mutations and altered cell permeability in colistin resistance in *Klebsiella pneumoniae* . Cells 11 (19), 2995. 10.3390/cells11192995 36230959 PMC9564205

[B121] Yeom J. Imlay J. A. Park W. (2010). Iron homeostasis affects antibiotic-mediated cell death in Pseudomonas species. J. Biol. Chem. 285 (29), 22689–22695. 10.1074/jbc.M110.127456 20479007 PMC2903419

[B122] Yu Z. Liu X. Du X. Chen H. Zhao F. Zhou Z. (2022). Pharmacokinetics/pharmacodynamics of polymyxin B in patients with bloodstream infection caused by carbapenem-resistant *Klebsiella pneumoniae* . Front. Pharmacol. 13, 975066. 10.3389/fphar.2022.975066 36588676 PMC9800617

[B123] Zavascki A. P. Goldani L. Z. Cao G. Superti S. V. Lutz L. Barth A. L. (2008). Pharmacokinetics of intravenous polymyxin B in critically ill patients. Clin. Infect. Dis. 47 (10), 1298–1304. 10.1086/592577 18840079

[B124] Zhang P. Qiu Y. Wang Y. Xiao L. Yu S. Shi M. (2022). Nanoparticles promote bacterial antibiotic tolerance via inducing hyperosmotic stress response. Small 18 (19), e2105525. 10.1002/smll.202105525 35398987

[B125] Zhao J. Han M. L. Zhu Y. Lin Y. W. Wang Y. W. Lu J. (2021). Comparative metabolomics reveals key pathways associated with the synergistic activity of polymyxin B and rifampicin combination against multidrug-resistant Acinetobacter baumannii. Biochem. Pharmacol. 184, 114400. 10.1016/j.bcp.2020.114400 33387481 PMC7906441

[B126] Zhao J. Zhu Y. Lin Y. W. Yu H. Wickremasinghe H. Han J. (2022). Polymyxin dose tunes the evolutionary dynamics of resistance in multidrug-resistant Acinetobacter baumannii. Clin. Microbiol. Infect. 28 (7), 1026.e1–1026.e5. 10.1016/j.cmi.2022.02.043 35283314

[B127] Zhu Y. Zhao J. Maifiah M. H. M. Velkov T. Schreiber F. Li J. (2019). Metabolic responses to polymyxin treatment in acinetobacter baumannii ATCC 19606: integrating transcriptomics and metabolomics with genome-scale metabolic modeling. mSystems 4 (1), e00157. 10.1128/mSystems.00157-18 30746493 PMC6365644

[B128] Zigrayova D. Mikusova V. Mikus P. (2023). Advances in antiviral delivery systems and chitosan-based polymeric and nanoparticulate antivirals and antiviral carriers. Viruses 15 (3), 647. 10.3390/v15030647 36992356 PMC10054433

